# Sleeve Gastrectomy and Roux-en-Y Gastric Bypass Alter the Gut-Brain Communication

**DOI:** 10.1155/2015/601985

**Published:** 2015-02-03

**Authors:** L. A. Ballsmider, A. C. Vaughn, M. David, A. Hajnal, P. M. Di Lorenzo, K. Czaja

**Affiliations:** ^1^Department of Veterinary Biosciences & Diagnostic Imaging, College of Veterinary Medicine, The University of Georgia, Athens, GA 30602, USA; ^2^Department of Neural & Behavioral Sciences, Penn State University, Hershey, PA 17033, USA; ^3^Department of Psychology, Binghamton University, Binghamton, NY 13902, USA

## Abstract

This study investigated the anatomical integrity of vagal innervation of the gastrointestinal tract following vertical sleeve gastrectomy (VSG) and Roux-en-Y gastric bypass (RYGB) operations. The retrograde tracer fast blue (FB) was injected into the stomach to label vagal neurons originating from nodose ganglion (NG) and dorsal motor nucleus of the vagus (DMV). Microglia activation was determined by quantifying changes in the fluorescent staining of hindbrain sections against an ionizing calcium adapter binding molecule 1 (Iba1). Reorganization of vagal afferents in the hindbrain was studied by fluorescent staining against isolectin 4 (IB4). The density of Iba1- and IB4-immunoreactivity was analyzed using Nikon Elements software. There was no difference in the number of FB-labeled neurons located in NG and DMV between VSG and VSG-sham rats. RYGB, but not RYGB-sham rats, showed a dramatic reduction in number of FB-labeled neurons located in the NG and DMV. VSG increased, while the RYGB operation decreased, the density of vagal afferents in the nucleus tractus solitarius (NTS). The RYGB operation, but not the VSG procedure, significantly activated microglia in the NTS and DMV. Results of this study show that the RYGB, but not the VSG procedure, triggers microglia activation in vagal structures and remodels gut-brain communication.

## 1. Introduction

Obesity is the largest nutrition-related condition affecting not only developed but also developing countries. It is a chronic and relapsing disease that is gaining prevalence among younger patients. Globally, over 400 million adults are clinically obese with a BMI of at least 30 kg/m^2^, and nearly 1.6 billion are overweight. In terms of therapeutic intervention, bariatric operations, including vertical sleeve gastrectomy (VSG) and Roux-en-Y gastric bypass (RYGB), are the most effective weight loss treatments for obese patients [1, 2]. Although the hormonal changes following VSG and RYGB have been well described [3] and are a major factor in the ensuing weight loss, the concomitant neural changes, underlying the antiobesity effect, remain unexplored.

Sensory information from the stomach is conveyed to the brainstem via gastric vagal afferents [4–7], the central terminals where the brainstem enters via the tractus solitarius and synapse on the nucleus tractus solitarius (NTS) neurons. The importance of visceral afferent signaling, via the “solitary-reticular ingestion system,” for the control of ingestive behavior has been previously established [8–11]. In this signaling system, cell bodies of vagal afferents are located in nodose ganglion (NG) [6] and approximately 70% of vagal afferents innervate the abdominal viscera, most notably the stomach and intestines [5, 12, 13]. A key function of abdominal vagal afferent signaling is participation in the control of food intake through responding to gastrointestinal stimuli [14–16]. The efferent innervation to the stomach originates from the dorsal motor nucleus of the vagus (DMV) [17–19] and the majority of DMV neurons project to the myenteric plexus, with the highest density of efferent fibers terminating in the stomach [17]. The stomach-hindbrain vagovagal circuit is comprised of sensory afferents terminating onto NTS neurons [5]. In turn, NTS neurons project to DMV cells to provide preganglionic control of cholinergic excitatory and NANC inhibitory postganglionic neurons [20]. Notably, recent reports indicate that sensitivity of vagal innervation to specific gastrointestinal stimuli is enhanced in obesity [21, 22] and after bariatric intervention [23]. However, much less is known regarding the reorganization of vagal innervation following the bariatric operation [24].

During bariatric procedures, gastric branches of the vagus nerve are cut by the gastrostomy technique creating damage to preganglionic efferent and afferent fibers [23, 25, 26]. However, there are significant differences between VSG and RYGB with regard to the location of the nerve cut. During VSG operation, the stomach is cut longitudinally [27, 28] and very distal branches of the gastric vagus are damaged, while in the RYGB procedure the stomach is cut transversely and gastric vagal branches are damaged very close to their origin from the esophageal plexus [29]. Therefore, it is quite likely that sensory input from the gastrointestinal (GI) tract, operating via the vagus nerve to selectively influence the food intake, may be altered after the bariatric operations [23]. Our recent studies support this hypothesis and indicate that subdiaphragmatic vagotomy triggers transient withdrawal and remodeling of central vagal afferent terminals in the NTS [30]. Moreover, damage to subdiaphragmatic vagal trunks triggers microglia activation in the dorsal vagal complex (DVC) of the hindbrain, a key structure that relays information from the GI tract to the CNS via the vagus nerve [31]. When viewed collectively, these observations strongly suggest that the beneficial and/or side effects of VSG and RYGB may be regulated in part, through alterations of anatomical integrity of vagal innervation between the hindbrain feeding centers and the GI tract. To test this hypothesis, the present study utilizes neuroanatomical approaches to assess damage to the GI innervation and reorganization of NTS following VSG and RYGB. The results of this effort provide the structural foundation for future functional investigations on the role of gut-brain communication following bariatric operation.

## 2. Materials and Methods

### 2.1. Animals

Male Sprague-Dawley rats (four-month old at the time of the operation, Simonsen Laboratories, Gilroy, CA, USA) were housed in individual hanging cages in a temperature-controlled vivarium with ad libitum access to standard rodent chow (Harlan Teklad F6 Rodent Diet W, Madison, WI, USA) and water. The rats were maintained on a 12-hour light/dark schedule. The rats were handled daily for a minimum of one week prior to the onset of experimental procedures. Rats were randomly assigned to four groups: sham/VSG, sham/RYGB, VSG, and RYGB (eight rats in each group). All animal procedures were approved by the Washington State University Institutional Animal Care and Use Committee and conform to National Institutes of Health Guide for the Care and Use of Laboratory Animals.

### 2.2. Operation

General anesthesia was used for all surgical procedures. Each rat was placed in a chamber and inhalation anesthesia (3% isoflurane, oxygen flow 2 L per minute) was delivered until the rats lost their righting reflex. The animal was maintained throughout the preparation and the operation period on inhalation anesthesia (on a scavenged mask circuit of isoflurane 1–3% to effect). Sterile procedures were followed in all operations. The animals were kept on a temperature-controlled surgical board (38°C) in dorsal recumbency. At the end of each operation, the retrograde tracer, fast blue (FB), was injected directly into the dorsal and ventral portion of the pylorus (1 *μ*L each injection) with a 5 *μ*L Hamilton syringe as previously described [6]. The needle was held in place for an additional one minute to ensure that the entire tracer was properly injected and to minimize leakage upon removal of the injector.

### 2.3. Vertical Sleeve Gastrectomy (VSG)

The VSG operation was performed as previously described [27]. Briefly, a midline abdominal incision was made extending about two-thirds the length of the abdomen to the xiphoid cartilage and a self-retaining retractor was placed. The liver was gently retracted cranially. Blunt dissection was carried along the greater curvature of the stomach and the greater curvature was freed from its attachments. Next, the lateral 80% of the stomach was excised using an ENDOPATH ETS 45 mm straight endocutter (Ethicon Endo-Surgery, Inc.). A sleeve was created along the lesser curvature preserving the gastroesophageal junction and the pylorus. The abdominal incision was closed with 3-0 PGA interrupted sutures in two layers (muscles and skin).

### 2.4. Roux-en-Y Gastric Bypass (RYGB)

The RYGB operation was performed as described before [32]. The day before operation, the rats were fasted overnight. On the day of operation, the rats were weighed and then anesthetized with isoflurane (3% for induction, 1.5% for maintenance). Ceftriaxone 100 mg/kg im (Roche, Nutley, NJ) was given as a prophylactic antibiotic. Under sterile conditions a midline laparotomy was performed. Next, the stomach was divided by using an ENDOPATH ETS 45 mm straight endocutter (Ethicon Endo-Surgery, Inc.). The staple line on the lesser curvature was placed two-three mm below the gastroesophageal junction. On the greater curvature, it was placed such that the resulting gastric pouch represented 20% of the original stomach size. The small intestine was divided to create a 15 cm biliopancreatic limb, a 10 cm alimentary (Roux) limb, and a 33 cm common channel. The gastrojejunal and jejunojejunostomies were performed by using interrupted 5-0 PGA sutures. Surgical incisions were injected with 0.5 mL of 0.25% bupivacaine to minimize postoperative discomfort. The abdominal incision was closed with 3-0 PGA interrupted sutures in two layers (muscles and skin). All rats were injected subcutaneously with normal saline (50 mL/kg, prior to the start of the operation, immediately after the operation, and again on postoperative day 1).

### 2.5. Sham Operation

Rats undergoing a sham operation were used as controls. The sham operation consisted of laparotomy and intestinal manipulation without stomach/gut resection followed by abdominal closure.

### 2.6. Postoperative Care

To allow the surgical anastomoses to heal, animals were not allowed to eat or drink until 24 h after the operation. For the following nine days (postoperative days 2–10), the rats were given Ensure liquid diet and water* ad libitum*. Sham-operated animals received the exact same postoperative care (including the one day of fasting and nine days of the maintenance diet).

### 2.7. Tissue Processing

Ten days after the operation, rats were anesthetized and transcardially perfused with 0.1 M phosphate-buffered saline (PBS; pH 7.4) followed by 4% paraformaldehyde in 0.1 M PBS. Hindbrains and NG were harvested, postfixed in 4% paraformaldehyde for two hours, and immersed overnight in 20% sucrose in PBS (pH 7.4). Hindbrains were sectioned at 30 *μ*m thickness throughout the rostrocaudal extent of the NTS (between bregmata −11.20 and −15.97 mm) and stained for selected antigens. For each studied region, tissue from all animals was processed simultaneously to prevent differences in staining due to differing conditions. Twenty *μ*m thick cryostat sections, of whole NG, were directly mounted in ProLong (Molecular Probes), to reduce photo bleaching, onto sets of four slides (total of 28–32 sections per ganglion; seven-eight sections per slide). Prior to staining, hindbrain sections were incubated for two hours in a blocking solution of 10% normal horse serum in trisphosphate buffered saline (TPBS, pH 7.4). One set of sections (*n* = 6 sections/hindbrain; evenly spaced throughout the rostrocaudal extent of the NTS) was then incubated in the primary antibody against IB4 (catalogue number I21414, Invitrogen; 10 *μ*L isolectin 4 in 3.99 mL PBS), used effectively for tracing central and peripheral nonmyelinated fibers [33]. IB4 is a 114 kDa glycoprotein and part of a family of five tetrameric type I isolectins isolated from the seeds of* Griffonia simplicifolia* [34]. Next, sections were incubated ExtrAvidin-CY3 (catalogue number E4142, Sigma Aldrich; 1 : 600) and mounted in ProLong (Molecular Probes). A separate set of sections (*n* = 6 sections/hindbrain; evenly spaced throughout the rostrocaudal extent of the NTS) was subsequently incubated overnight in a primary antibody against an ionized calcium binding adapter molecule 1 (Iba1; rabbit polyclonal, 1 : 1000; catalogue number 019-19741, Dako) followed by an Alexa-488 secondary antibody (donkey anti-rabbit, 1 : 400; catalogue number A21206, Invitrogen). Iba1 was used as a marker of activated microglia [31]. The primary antibody against Iba1 recognizes a single band at 17 kD on western blots from adult rat spinal cord homogenates [35]. To determine whether nonspecific staining from the secondary antibody was present, an additional control group was included in which the primary antibody was omitted and replaced by a preimmune serum. Both the preabsorption and the omission abolished immunostaining for both antisera. Sections were mounted in ProLong (Molecular Probes) to reduce photo bleaching.

### 2.8. Neuron Counts and Density Analysis

Sections were examined under a Nikon 80-I fluorescent microscope. FB-labeled neurons in NG and DMV were counted in every fourth section (total seven-eight sections per ganglion) to eliminate the likelihood of counting the same neuron twice. The area fraction of Iba1 and IB4 immunofluorescence was analyzed using Nikon Elements AR software as previously described [30, 36]. For each studied region, a representative section from each animal was used to calculate an average exposure time and background fluorescence level as determined by the pixel intensity of stained tissue regions that were negative for Iba1. Subsequently, 20x-stitched images of the hindbrain (*n* = 6 sections/hindbrain; evenly spaced throughout the rostrocaudal extent of the NTS) were created using this fixed/standardized exposure time followed by the removal of background fluorescence. In hindbrain sections, regions of interest (ROIs) were created to isolate the NTS and DMV from one another. The resulting data are expressed as mean ± SEM and were analyzed using a one-way ANOVA followed by a Holm-Sidak test for significance.

## 3. Results and Discussion

Results of the study indicate that both the VSG and RYGB procedures damage the vagal innervation of the stomach. However, the nature of this damage and subsequent consequences on hindbrain signaling circuits differ between each procedure. This neuroanatomical observation from our study is supported by prior investigations and is reviewed by Stefater and collaborators [3]. Our previous studies revealed that the significant withdrawal of vagal afferents from the hindbrain was observed at 10 days after vagotomy [30]. Moreover, the most significant impact of a bariatric operation on body weight loss in rats is observed in the first two weeks after the procedure [27, 37]. Therefore, in the present study, vagal gut-brain communication was investigated 10 days after VSG and RYGB. Because obesity was previously reported to induce changes in vagal gut-brain communication [38–40], the present study was performed on lean rats to avoid additional variable affecting hindbrain reorganization.

**Figure 1:**
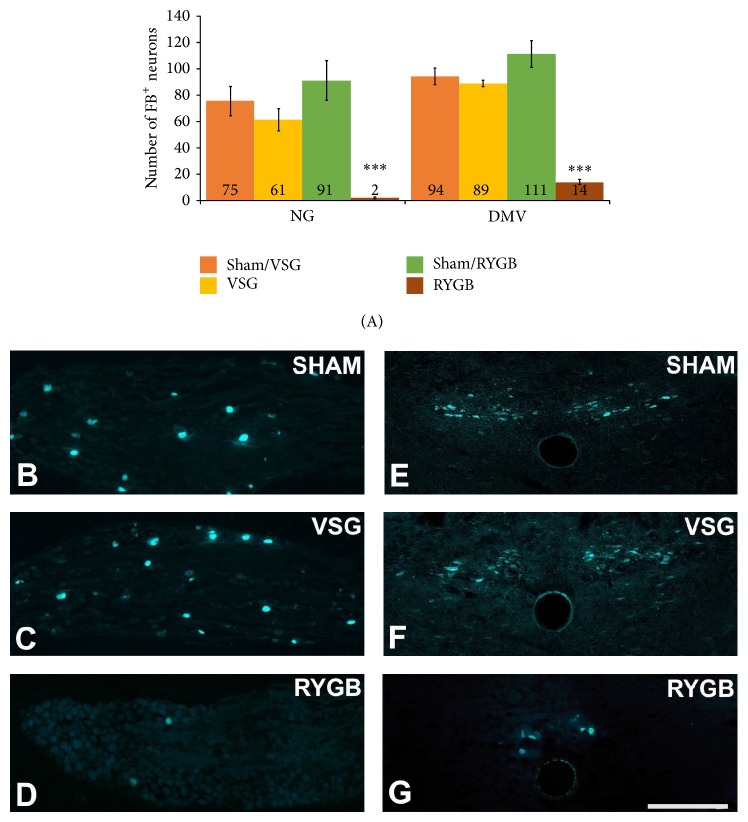
The RYGB operation dramatically reduced the number of retrogradely labeled (FB) afferent ((A), (D) in NG) and efferent ((A), (G) in DMV) neurons innervating the stomach. We found no significant difference in the number of afferent and efferent FB-labeled neurons innervating the stomach following sham ((A), (B) and (A), (E), resp.) or VSG ((A), (C) and (A), (F), resp.) surgery. (B)–(D) Representative sections of NG: nodose ganglion showing FB-labeled neurons. (E)–(G) Representative sections of DMV: dorsal motor nucleus of the vagus showing FB-labeled neurons. All data are expressed as average ± SEM; *P* < 0.001; scale bar = 200 *μ*m.

In our study, we used a retrograde tracer fast blue (FB) to reveal changes in stomach-hindbrain communication following VSG and RYGB. We found no significant difference in the number of afferent (75 ± 11 versus 61 ± 8; NG) and efferent (94 ± 6 versus 89 ± 2; DMV) FB-labeled neurons innervating the stomach following the sham or the VSG operation (Figure 1). In contrast, after RYGB, the number of FB-labeled neurons in both the NG (2 ± 0.6) and DMV (14 ± 2) was dramatically reduced in comparison to sham-operated rats (91 ± 15 and 111 ± 10, resp.; Figure 1). The dorsal and the ventral gastric branches of the subdiaphragmatic vagus provide the majority of the vagal innervation to the stomach [41], and these branches are transected during the VSG and RYGB procedure [23]. However, the location of the cut with respect to these branches is different in VSG and RYGB. During VSG, the stomach is divided longitudinally and only very distal fibers of gastric branches are severed while, during RYGB, the stomach is transected transversely and both the ventral and the dorsal branches of the gastric vagus are transected [42]. The very few retrogradely labeled neurons found after RYGB in NG and DMV (Figure 1) may represent the previously reported collateral projections to the stomach from celiac and accessory celiac branches of the subdiaphragmatic vagus [43], which are spared during the RYGB procedure. Nonetheless, the implication of this result is that RYGB but not VSG compromises the retrograde transport via both the afferent and the efferent fibers of the gastric vagal branches and disconnects the vagal signaling from the stomach to the hindbrain. It is important to note here that the stomach receives the spinal innervation via the splanchnic nerve [44, 45] and that the spinal innervation of the stomach may undergo compensatory plasticity after RYGB to provide an alternative pathway for stomach/brain communication. This hypothesis is supported by our previous studies which revealed vagotomy-induced changes in microglia activation in the thoracic and lumbar segments of the spinal cord [31]. However, further investigations are necessary to establish the role of splanchnic innervation in gut-brain communication and the functional role of this process following bariatric operations.

**Figure 2:**
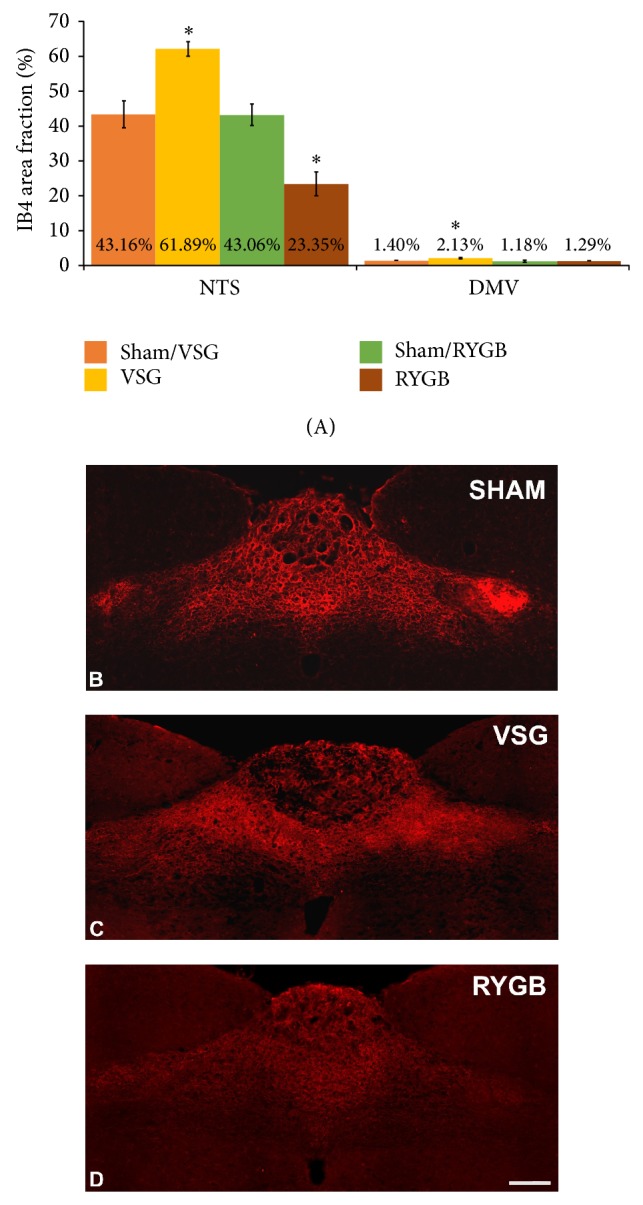
The VSG operation increased the density of vagal afferent input into the NTS and DMV (A), (C) with respect to sham-operated controls (A), (B). The RYGB operation decreased the density of vagal afferent input into NTS with no changes in DMV. (B)–(D) Representative coronal sections of the intermediate NTS revealing IB4-immunoreactive fibers. All data are expressed as average ± SEM; *P* < 0.05; scale bar = 200 *μ*m.

We also analyzed the density of vagal afferents in the NTS and DMV to reveal a reorganization of vagal circuits in the hindbrain after VSG or RYGB. Our results indicate that VSG significantly increased the density of vagal afferents in the NTS and the DMV (61.89% ± 2.03% and 2.13% ± 0.21%, resp.) when compared to sham-operated controls (43.16% ± 3.82% and 1.40% ± 0.05%, resp.; Figure 2). The RYGB operation, in turn, significantly decreased the density of vagal afferents in the NTS (23.35% ± 3.44% versus 43.06% ± 3.05%) and produced no change in the DMV (1.29% ± 0.13% versus 1.18% ± 0.32%; Figure 2) relative to controls. Excitatory input from the stomach to the NTS is conveyed via vagal afferents to selectively influence the satiety and regulate food intake [14, 15, 46]. Therefore, the reorganization of the feeding centers in the hindbrain after VSG and RYGB likely plays a functional role in the antiobesity effect of bariatric operations. It is necessary to note here that although both operations lead to sustained weight loss, the effects of each manipulation on hindbrain reorganization are very different. The effect produced by VSG resembles a sprouting or thickening of central neurites, previously reported in the spinal cord after sciatic nerve transection [47, 48]. VSG-induced remodeling could lead to inappropriate hindbrain responses (over excitation) to gastric stimuli. In similar studies, using the somatosensory model injury-induced sprouting contributes to the pathophysiology of allodynia, in which light touch sensations are perceived as painful stimuli [49–51]. Thus, the increased sprouting of hindbrain neurons observed after VSG may reflect a sensitized functional response to food related stimuli from the gut.

The reduced density of vagal afferents after RYGB, observed in our study, may reflect the withdrawal of vagal afferents from the NTS observed previously after subdiaphragmatic truncal vagotomy [30]. Truncal vagotomy also produced transient decreases in spontaneous glutamate release, glutamate release probability, and the number of primary afferent inputs [30]. Interestingly, several studies report that vagotomy reduces food intake and body weight [52–54]. Moreover, recent studies indicate that signals carried by vagal afferents from the GI tract contribute to the early RYGB-induced body weight loss and reduction of food intake [26]. Both the previously published studies and results of the current study show that VSG produces minor damage to the gastric vagus and the antiobesity effect of this operation depends mostly on the restrictive nature of this operation [55–57]. However, the restrictive dogma of VSG has been challenged by recent data from both humans and rodents [58]. Although the hormonal changes following RYGB have been well described and are a major factor in the ensuing weight loss [59–61], the concomitant neural changes, underlying the effects on food intake, are still not completely understood [23]. Results of our study provide a deeper understanding of the role of the neural component in the mechanism of RYGB and show that damage of vagal innervation, to the stomach, produces reorganization of feeding centers in the brain.

**Figure 3:**
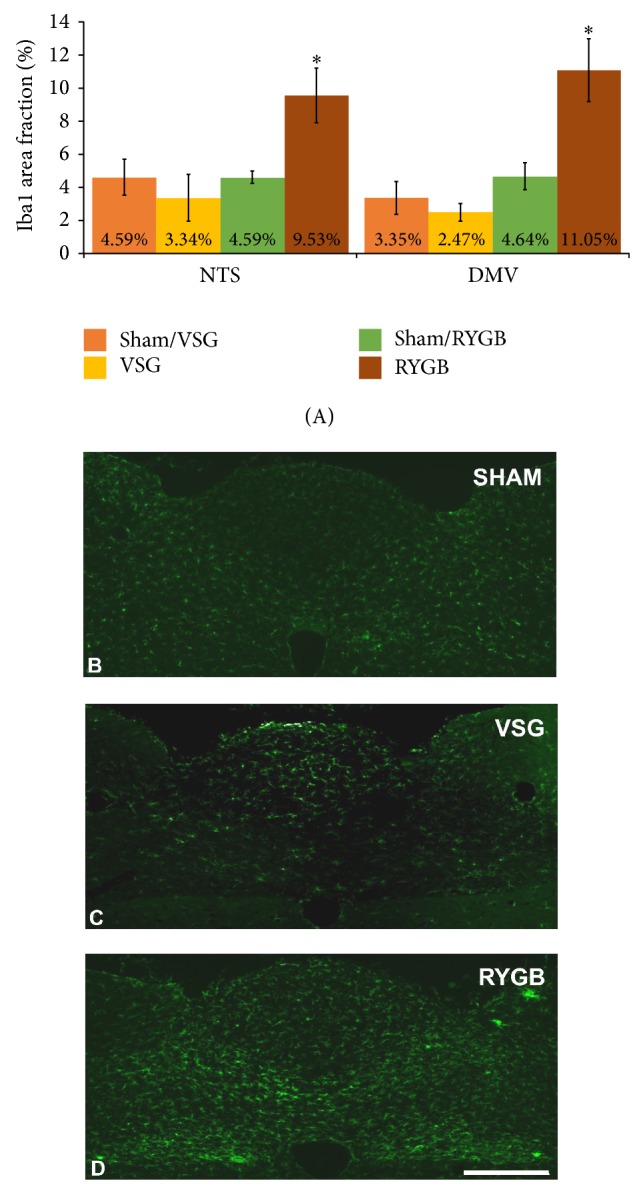
The RYGB operation induced activation of microglia in the NTS and DMV (A), (D). There were no differences in the microglia activation between VSG (A), (C) and sham-operated controls (A), (B). (B)–(D) Representative coronal sections of the intermediate NTS revealing Iba1-immunoreactive microglia. All data are expressed as average ± SEM; *P* < 0.05; scale bar = 200 *μ*m.

In the last part of our investigation, we tested the hypothesis that VSG and RYGB would result in microglia activation in the NTS and DMV. Our results show that, after a sham operation, the studied hindbrain nuclei contained Iba1-immunoreactive microglia with resting morphology. This resting morphology was reflected by cells with small perikarya and radially branching processes (Figure 3). The RYGB operation significantly activated microglia in the NTS and DMV (9.53% ± 1.64% and 11.05% ± 1.91%, resp.) when compared to sham-operated controls (4.59% ± 0.36% and 4.64% ± 0.81%, resp.; Figure 3). The VSG procedure, in turn, did not increase the microglia activation within the studied hindbrain centers (Figure 3). It has been previously reported that subdiaphragmatic truncal vagotomy [31] and unilateral NG removal [62, 63] activated microglia and increased inflammatory markers in the NTS and DMV. This activation of microglia may have an effect on vagal structures through cytokine release because the cytokine IL-1*β* has been shown to activate vagal afferents in the NG [64]. Furthermore, IL-1*β* and TNF-*α* have been shown to play a role in decreased food intake, decreased gastric motility, and increased lipid metabolism. However, the role of cytokines in the antiobesity effect of bariatric procedures needs further investigations.

## 4. Conclusions

In summary, our results and the previous studies show RYGB-induced damage to vagal innervation similar to truncal subdiaphragmatic vagotomy [30, 31]. This damage may be reflected by a long-term reorganization of hindbrain feeding centers (NTS and DMV) and decreased vagal input as well as glutamate release in the NTS. However, further functional studies aimed at glutamate release following RYGB are required to challenge this hypothesis. RYGB-induced activation of microglia points to a plausible mechanism whereby surgically induced cytokine release in the hindbrain feeding centers would lead to decreased food intake and reduced body weight. However, the role of cytokines in the antiobesity effect of bariatric procedures needs further investigations. Results of the study also show that the VSG operation is less invasive to the vagal innervation of the GI tract than RYGB. It produced sprouting of vagal afferents synapsing in the NTS without long-term inflammatory responses. This sprouting of vagal inputs raises the possibility that VSG induces hyperexcitation of NTS synapses and increased glutamate release. Consequently, hyperexcitation of hindbrain feeding circuits could be a functional mechanism regulating decreased food intake and subsequent body weight loss. Future studies are required to fully describe this phenomenon.

In conclusion, the results show that both RYGB and VSG may induce metabolic improvements via different neural mechanisms. To advance our understanding of these procedures, it is necessary to determine the long-term changes in gut-brain communication following bariatric operations. This will allow for greater mechanistic insight into how a bariatric operation results in sustained weight loss and improved eating behaviors and, in turn, facilitates the development of novel antiobesity treatments that could achieve comparable weight loss, without surgical risks.

## Abbreviations

DMV: Dorsal motor nucleus of the vagus
DVC: Dorsal Vagal Complex
FB: Fast blue
GI: Gastrointestinal
IB4: Isolectin 4
Iba1: Ionizing calcium adapter binding molecule 1
NG: Nodose ganglion
NTS: Nucleus tractus solitarius
PBS: Phosphate-buffered saline
ROIs: Regions of interest
RYGB: Roux-en-Y gastric bypass
TPBS: Trisphosphate buffered saline
VSG: Vertical sleeve gastrectomy.
